# Strengthening Outbreak Detection in Africa to Achieve the 7-1-7 Global Framework: Challenges and Opportunities

**DOI:** 10.3389/phrs.2025.1608039

**Published:** 2025-08-14

**Authors:** Frantz Jean Louis, Lisa Nichols, Cristina de la Torre, Anicet G. Dahourou

**Affiliations:** ^1^ ICF-Infectious Disease Detection and Surveillance Project of USAID, Rockville, MD, United States; ^2^ FHI 360, Washington, DC, United States

**Keywords:** outbreak detection, diagnostic network, surveillance, global health, infectious disease

## Abstract

**Objectives:**

Timely detection of infectious disease outbreaks is essential to limit health, social, and economic impacts, yet diagnostic and surveillance gaps persist across Africa. This review applies the 7-1-7 global target framework—detect within 7 days, notify within 1, and respond within 7—to assess strategies for strengthening early detection capacities across African countries.

**Methods:**

We conducted a review of peer-reviewed literature, institutional reports, and field evidence published without time span limitations. Key themes were organized around five strategic pillars: diagnostic preparedness, surveillance, workforce development, community engagement, and governance.

**Results:**

Identified bottlenecks include limited diagnostic networks capacity, fragmented surveillance systems, workforce shortages, and underinvestment in digital infrastructure. Promising solutions include diagnostic network optimization, deployment of point-of-care molecular tools, integration of event- and indicator-based surveillance through interoperable platforms, and AI-enabled early warning systems. Field examples from Uganda, Senegal, and Nigeria demonstrate improved timeliness where coordinated investments and multisectoral collaboration have been implemented.

**Conclusion:**

Meeting the 7-1-7 detection target requires integrated, country-owned strategies that align diagnostics, surveillance, workforce, and governance within resilient national health security frameworks, underpinned by sustained domestic investment.

## Introduction

In 2023, the African continent faced a staggering eighty major public health events and sixty-one outbreaks [1]. Given the constraints in many resource-limited countries, strengthening their capacities for early detection, notification, and response is paramount to mitigating the impact of public health emergencies. Rapid identification and effective response play a pivotal role in minimizing morbidity and mortality during such critical events. The capacities for early detection, notification, and response to emerging infectious diseases are defined by the legally binding International Health Regulations 2005 (IHR 2005). These regulations require the 196 signatory member states to prevent, protect against, control, and provide a public health response to the international spread of diseases [2].

Outbreak detection refers to the initial identification of a potential public health threat, typically through surveillance systems that monitor disease patterns in populations. However, detection alone is not sufficient; confirmation of the outbreak is equally critical. Confirmation involves verifying the presence of the disease through laboratory testing or other diagnostic methods to ensure that the detected signal indeed represents a threat. Both detection and confirmation are essential for timely and accurate outbreak response [3]. Early detection allows for rapid public health interventions, while confirmation ensures that resources are appropriately allocated to real threats, preventing unnecessary panic and optimizing the response strategy.

Timeliness in detection and response remains a critical aspect of robust surveillance systems. However, poor diagnostic preparedness has significantly contributed to delays in identifying recent outbreaks. The insufficient testing capacity and delays in testing and reporting exacerbated the expansion of the 2013–2016 Ebola epidemic in West Africa [4]. A three-month delay occurred between the index case and the identification of the causative agent [5, 6]. Post-outbreak analyses reveal that diagnosing 60% of patients within 1 day of symptoms onset instead of 5 days could have reduced the attack rate from 80% to nearly 0% [4, 7, 8]. Although timeliness metrics (such as time to detection, time to verification, time to response) do not fully capture the complexity of outbreak surveillance and response, they have been proposed as valuable tools for monitoring and evaluating outbreak response performance, with the aim of optimizing response efforts [9, 10].

Resolve to Save Lives proposed a 7-1-7 that sets three time-bound, real-world performance targets across the entire outbreaks detection-to-response continuum: ≤7 days from a pathogen’s emergence to detection of a suspected outbreak, ≤1 day from detection to formal notification and initiation of investigation, and ≤7 days from notification to completion of essential early-response actions (e.g., case confirmation, contact tracing, risk communication, infection-control measures) [11, 12]. Because these milestones are simple, measurable, and pathogen-agnostic, they expose bottlenecks anywhere in the surveillance-response chain and allow rapid, data-driven performance improvement—much as the HIV 90-90-90 targets accelerated antiretroviral scale-up [11, 13]. Recognizing its value, the World Health Organization (WHO) embedded 7-1-7 metrics in its 2023 *Early Action Review* (EAR) guidance as the primary yard-stick for rapid performance enhancement during health emergencies [14]. EAR promotes structured, rapid learning from recent public health events to inform timely improvements in detection systems before the next crisis emerges [15]. By conducting routine timeliness-focused reviews after major outbreaks, countries can systematically assess bottlenecks across the surveillance and diagnostic continuum. Integrating EARs into national outbreak response strategies can help institutionalize continuous learning, improve timeliness metrics, and align response systems with the 7-1-7 targets. The WHO Regional Office for Africa (WHO AFRO) has officially adopted the 7-1-7 indicators as a key target for timeliness in its 2022–2030 Regional Strategy for Health Security and Emergencies [16].

To improve outbreak detection and confirmation capacity and timeliness, a comprehensive surveillance system across human, animal, and environmental health (ecosystem) sectors should be implemented. Robust governance is necessary to ensure that these elements are well coordinated, adequately resourced and aligned with national and international standards. Here we outline strategies for African countries to achieve this seven-day detection target. The strategies are built on five main pillars:

Enhance diagnostic preparedness: Invest in laboratories and diagnostic networks for rapid pathogen identification.

Strengthen surveillance: Develop robust disease surveillance systems to detect outbreaks early, leveraging both traditional and more proactive surveillance approaches.

Develop multisectoral public health workforce: Train healthcare workers in diagnostics and outbreak response.

Engage Communities: Involve communities in outbreak detection and response.

Strengthen Governance: Establish and enforce clear policies for effective outbreak detection and response.

Through the adoption of these comprehensive strategies, countries can build a more resilient public health infrastructure, capable of not only detecting outbreaks early but also responding swiftly and effectively.

## Methods

This review was conducted to synthesize evidence on strategies, challenges, and opportunities for achieving the early detection target of the 7-1-7 framework in African countries. A comprehensive search was done, focusing on peer-reviewed literature, grey literature, and field-based programmatic reports published in English without time span limitations. We systematically searched PubMed, Scopus, and Google Scholar using combinations of keywords such as “7-1-7 framework,” “outbreak detection,” “surveillance,” “diagnostic preparedness,” “diagnostic networks” “workforce development,” “laboratory governance,” “event-based surveillance,” and “indicator-based surveillance.” References cited in key studies were also reviewed to identify additional relevant publications. WHO, the Africa Centres for Disease Control and Prevention (Africa CDC), the United States Agency for International Development (USAID)’s Infectious Disease Detection and Surveillance (IDDS) project reports, and other institutional documents were reviewed to supplement findings, particularly for field examples and implementation case studies. The review highlights thematic areas aligned with the five strategic pillars: enhancing diagnostic preparedness, strengthening surveillance systems, developing a multisectoral workforce, engaging communities, and strengthening governance. Findings were analyzed and summarized narratively, illustrating key strategies to address bottlenecks and support countries to meet the seven-day detection milestone of the 7-1-7 global framework.

## Results

### Enhance Diagnostic Preparedness

Diagnostics play a crucial role in successful outbreak detection and containment. When outbreaks occur, timely and accurate diagnosis is essential for identifying affected individuals, confirming the pathogenic agent, implementing control measures, and preventing further spread. Unfortunately, recent outbreaks of pathogens like Ebola, Zika, and yellow fever have highlighted challenges in diagnostic preparedness, including limited access to reliable tests and inadequate testing capacity [4, 7, 17, 18]. These gaps can lead to delays in detection, surveillance, and containment, ultimately exacerbating the impact of outbreaks [7]. A structured three-step cycle—assess → optimize → implement—can provide a practical roadmap for countries to build resilient, outbreak-ready diagnostic networks. The first step to improve diagnostic preparedness is to assess the current capacity of the diagnostic network for surveillance and outbreak response.

#### Diagnostic Network Assessment

Diagnostic network assessment (DNA) provides an opportunity to comprehensively evaluate its readiness to timely and accurately detect emerging infectious disease threats. The assessment should include governance and policies, laboratory infrastructure, human resources, biosafety/biosecurity, quality management, data management, and use of diagnostic technologies across the country. Many assessment tools are available to help review laboratories’ capacities [19, 20], but these tools focus on specific diseases or individual laboratories and may not be aligned with a comprehensive “One Health” approach that integrates the ecosystem health sectors. To address this gap, the USAID’s IDDS project (2018–2024), developed a new comprehensive diagnostic network assessment tool to evaluate an entire diagnostic network’s ability to detect and effectively respond to emerging disease threats [21].

#### Diagnostic Network Optimization

Once gaps are identified, necessary steps need to be taken to improve the diagnostic network. Effective diagnostic networks integrate equipment, infrastructure, and human resources to ensure timely and accurate disease diagnosis [22, 23]. By optimizing resource allocation and enhancing access to high-quality testing, countries can respond effectively to outbreaks. Diagnostics Network Optimization (DNO) is a geospatial analytics approach that leverages geographic and health data to analyze the existing diagnostic network within a country or region [22–24]. By considering disease burden, health system capacity, and infrastructure, DNO aims to develop networks that facilitate optimal access to testing for patients. It recommends the strategic placement of diagnostic equipment, ensuring that areas with high disease prevalence have specialized testing facilities, while regions with lower burden focus on basic point-of-care testing [25, 26]. Multiple tools are available to conduct DNO. An open-access web-based tool called OptiDx is used to support DNO analysis [27]. In addition, geospatial analytics tools like AccessMod and LabMap/PlanWise offer complementary insights into the accessibility of diagnostic services [28, 29].

#### Priority Upgrades and Expansion

##### Upgrade Regional & Reference Laboratories

Investments to enhance the efficiency of diagnostic networks for detecting epidemic-prone pathogens should be guided by each country’s priority pathogen list and tailored to the most appropriate diagnostic tools and algorithms for each target pathogen. Molecular technologies play a pivotal role in early detection of diseases. These technologies provide critical insights based on the exploration of pathogens genomes. Real-Time Polymerase Chain Reaction (PCR) allows rapid and precise detection of pathogens’ DNA or RNA sequences. Other molecular technologies, such as loop-mediated isothermal amplification (LAMP) and enzyme-linked immunosorbent assay (ELISA) have also contributed to improving infectious disease detection and surveillance. Next-generation sequencing (NGS) allows for the rapid sequencing of part or the entire genomes, providing a comprehensive understanding of the genetic makeup of pathogens. In addition to PCR and NGS, other molecular technologies, such as CRISPR/Cas9 CRISPR/Cas9-based diagnostic tools have emerged as powerful and versatile platforms for detecting infectious diseases, offering high sensitivity, specificity, and speed [30, 31]. Many of these platforms are being adapted for use in resource-limited settings and point-of-care applications, which could have a significant impact on global health.

##### Expand Diagnostic Capacity Closer to Communities

Bringing diagnostic services closer to communities enhances timely access to diagnostic testing and improves patient care. The development of flexible, multi-pathogen diagnostic platforms is becoming particularly valuable for initial detection and monitoring of outbreak-causing pathogens, allowing rapid response without the need of extensive human resources and training requirements [4, 32]. One example of such a platform is GeneXpert. GeneXpert enables rapid and accurate detection of pathogens, including Ebola, COVID-19, Mpox, and other infectious diseases. GeneXpert can be deployed at the community level, eliminating the long turnaround time experienced at centralized laboratories and allowing timely intervention and containment during outbreaks [33, 34].

Rapid Diagnostic Tests (RDTs) also play a crucial role in outbreak detection by enabling the quick identification of infectious diseases, particularly in remote settings. RDTs are portable, easy-to-use, and require minimal infrastructure, allowing healthcare workers to bring diagnostic capabilities directly to affected communities [35]. During the Ebola outbreak in West Africa (2014–2016), RDTs were vital for early detection of cases, helping to curb the spread of the virus [36, 37]. Antigen-detection RDTs have been deployed on an unprecedented scale, including in non-healthcare settings, to help curb the spread of SARS-CoV-2 [38]. However, a significant challenge is that many diseases with epidemic potential, such as some forms of viral hemorrhagic fevers, do not have approved RDTs. Developing RDTs for a broader range of pathogens is essential for improving global outbreak preparedness and response [39].

##### Strengthen Specimen Transport and Referral System

A robust specimen referral system (SRS) is pivotal in expanding access to diagnostic services, particularly in regions where advanced laboratory facilities are limited or absent. By facilitating the efficient transport of clinical samples from peripheral health centers to centralized laboratories with advanced diagnostic capabilities, SRS ensures that patients in remote or resource-limited areas can benefit from accurate and timely diagnosis [40, 41]. This is especially crucial during outbreaks, where rapid detection of pathogens is essential for effective response. For instance, during the COVID-19 pandemic, referral systems were crucial in ensuring that samples from underserved areas were tested in well-equipped facilities, allowing for comprehensive surveillance, and effective response efforts [42, 43].

### Strengthen Surveillance

Establishing robust surveillance and response systems for early detection and rapid response to emerging zoonotic disease outbreaks is crucial in preventing epidemics, reducing their impact and financial burden [44]. The Integrated Disease Surveillance and Response (IDSR) regional strategy is a key foundational tool used to strengthen national public health surveillance and response systems at the national, district, health facility and community levels [45]. The IDSR strategy incorporates both Indicator-Based surveillance (IBS) and Event-Based Surveillance (EBS) approaches to early detection of priority diseases, conditions and events [45].

#### Traditional Surveillance Approaches

The third edition IDSR Technical Guidelines for the African Region (2019) reorganized national surveillance architecture into two complementary domains: IBS and EBS [45]. IBS is defined as the systematic collection, analysis, and interpretation of structured data based on predefined case definitions and reporting formats [45]. IBS forms the foundation of routine surveillance and is used for tracking trends and thresholds of notifiable diseases [45]. EBS is defined as the organized collection, monitoring, assessment, and interpretation of unstructured information regarding events that may pose a risk to public health [45]. EBS is designed to rapidly detect unusual health events or outbreaks that may not be captured through structured IBS systems [45]. Traditional IBS, e.g., mandatory disease-specific notification, laboratory-based surveillance, and syndromic surveillance, has been complemented by event-based surveillance (EBS), which gathers and analyzes information from various sources formal or informal, including media reports, rumors, and social media [46]. For example, in 2018, Nigeria experienced a major Lassa fever outbreak. In response, the Nigeria Centre for Disease Control (NCDC) activated its Incident Management System, enhanced its IBS, and formally integrated EBS into the national disease surveillance strategy [47, 48]. These strengthened systems enabled the timely detection of an increased case density and broader geographic spread of Lassa fever across multiple states between 2018 and 2020 [47, 48].

However, these traditional approaches have limitations, including lack of sensitivity and threshold dependence of IBS: predefined thresholds may miss emerging outbreaks with subtle increases and some outbreaks may not trigger alerts due to slow accumulation of cases [49]. For instance, during the early stages of the COVID-19 pandemic, traditional IBS systems were slow to detect the novel coronavirus, delaying global response efforts [50]. While EBS can detect unusual events that IBS might miss, it often suffers from issues such as false positives, data overload, and difficulties in distinguishing between significant health threats and benign events [51].

#### One Health Surveillance

In addition to traditional methods, there is a need to integrate surveillance of zoonotic diseases in human and animal populations in the context of One Health, for an effective response to diseases transmitted between animals and humans [52]. Environmental health surveillance is also an important component in predicting future outbreaks by monitoring environmental risk factors [53, 54]. In 2019, the Food and Agriculture Organization of the United Nations, the World Organization for Animal Health, and WHO developed the Tripartite Zoonoses Guide to support global health security efforts for zoonoses surveillance in a coordinated manner [55]. Health surveillance systems rely upon data from a variety of sources, often siloed, thereby causing inefficiencies and sub-optimal performance. By combining data from the ecosystem health, surveillance systems can identify emerging diseases, detect spillover events, and understand the complex interactions between human and animal populations. Kenya has established a robust One Health surveillance system through its Zoonotic Disease Unit (ZDU), a joint coordination mechanism between the MoH and Ministry of Agriculture created in 2011 [56]. The ZDU, in collaboration with partners, has deployed county-level One Health teams in over 30 counties to strengthen multisectoral reporting and response to zoonotic diseases [56]. Over a decade of capacity building (2006–2016) has led to the integration of animal and human health surveillance and the joint training of medical and veterinary field epidemiologists [56]. This approach has resulted in faster outbreak detection, coordinated investigations, and improved containment of infectious disease threats [56].

#### Innovation in Infectious Diseases Surveillance Systems

##### Artificial Intelligence Enhanced Surveillance/Big-Data Analytics

The integration of artificial intelligence (AI) and machine learning (ML) can significantly enhance the capabilities of traditional surveillance systems by enabling real-time analysis of vast amounts of data from diverse sources [57]. Big data analytics can sift through electronic health records, prescription data, internet searches, and social media activity to identify patterns that may indicate the emergence of an outbreak [58]. AI and ML can also improve the precision of EBS by reducing false positives and enhancing the ability to detect subtle signals that may indicate the early stages of an outbreak [59]. Data integration and analysis from multiple sources is a core component of an effective infectious disease Early Warning Systems [60]. For example, AI algorithms were instrumental in providing early warnings for the COVID-19 outbreak; systems like BlueDot and EPIWATCH used AI to scan global news reports and other open-source data to detect early signs of the virus spreading [61]. Internet-based Early Warning Systems carry huge potential for outbreak detection by enhancing communication across surveillance networks [62]. The Program for Monitoring Emerging Diseases (ProMED) is a pioneer in the field of digital disease surveillance. ProMED collates information from formal and informal sources including media reports, official reports, social media, local observers, and a network of clinicians throughout the world to detect oubreaks [62, 63]. HealthMap is another widely used tool for disease outbreak monitoring, that utilizes online news aggregators, eyewitness reports and other formal and informal sources of information for alerts visualization [64].

##### Wastewater Based Epidemiology Surveillance

Beyond reactive approaches to detect outbreaks, more proactive innovative strategies can enhance preparedness and minimize the impact of outbreaks. Wastewater based epidemiology (WBE) surveillance can be used as a complementary surveillance tool to provide rapid and reliable information to communities about diseases circulation and help to monitor disease outbreaks. By tracking pathogens concentration and diversity in wastewater, WBE allows early detection of outbreaks, and can provide information on outbreak location and hotspots [65, 66]. WBE surveillance data can be integrated with dynamic within-host and between-host models, or coupled with AI offering a novel, and proactive approach to predicting outbreaks [67–69]. However, challenges remain, including population mobility, pathogen detection sensitivity, and the standardization of testing methods [70].

##### Genomic Surveillance for Real-Time Pathogen Characterization

Genomic surveillance, the systematic sequencing of pathogen genomes and real-time sharing of the data, has become an essential complement to indicator- and event-based systems [71]. Whole-genome sequencing (WGS) enables rapid species confirmation, antimicrobial-resistance profiling, and phylogenetic tracing that links scattered cases into a single outbreak [72]. Genomics combined with machine-learning analytics consistently shortened time to source identification and improved containment in healthcare and community settings [73]. Large-scale, near-real-time sequencing of SARS-CoV-2 demonstrated the public-health value of tracking variants to inform vaccine updates and non-pharmaceutical interventions [74]. Frameworks published in 2024 outline stepwise approaches for embedding pathogen genomics in routine surveillance, emphasizing workforce training, data-sharing governance and integration with electronic IDSR platforms [72].

### Workforce Development

Workforce development for outbreak detection and response is a multifaceted challenge that extends beyond training and capacity building. It requires ensuring workforce retention, leveraging public-private partnerships and fostering interdisciplinary collaboration. Key successful strategies include the role of training programs like the Field Epidemiology Training Program (FETP), Africa CDC–Regional Integrated Surveillance and Laboratory Networks (RISLNET), the One Health Workforce (OHW) Program and the Global Outbreak Alert and Response Network (GOARN) Training, the Global Laboratory Leadership Program (GLLP) among others.

#### Field Epidemiology Training Programs (FETP)

Since 1975, the US Centers for Disease Control and Prevention has worked with countries worldwide to develop the FETP [75]. FETP are pivotal in strengthening the public health workforce through hands-on, field-based training in epidemiology, surveillance, and outbreak investigation [75]. Under the mentorship of experienced professionals, FETP trainees gain critical skills in outbreak detection and response, often taking on leadership roles within national public health institution [76]. As of 2024, around 98 FETPs exist worldwide, producing over 20,000 graduates; most trainees remain in their home countries, bolstering national public health capacity [77].

#### One Health Workforce (OHW) Program

The COVID-19 pandemic highlighted the urgent need for professionals who can address health challenges at the ecosystem interface [78, 79]. The USAID One Health Workforce -Next Generation (OHW-NG), officially launched in October 2019, built upon the foundations of the earlier One Health Workforce project (2014–2019) to train a new generation of health professionals equipped to tackle emerging global health threats [80, 81]. Since its inception, OHW-NG has engaged participants from over 90 countries, and has trained more than 4,000 individuals through various courses [81]. In collaboration with the Africa One Health University Network (AFROHUN)—formally launched in October 2020—and the Southeast Asia One Health University Network (SEAOHUN)—established in late 2011—OHW-NG supports the implementation of scalable and sustainable training systems [82, 83]. These efforts aim to strengthen interdisciplinary collaboration in outbreak prevention, detection, and response [84].

#### Africa CDC–Regional Integrated Surveillance and Laboratory Networks (RISLNET)

RISLNET is an initiative by Africa CDC, launched in 2019, to strengthen disease surveillance and laboratory networks across Africa. The program focuses on training and capacity building, fostering collaboration among countries in outbreak detection and response [85]. RISLNET has played a key role in enhancing regional collaboration during outbreaks, such as the Ebola and COVID-19 responses, by improving laboratory and surveillance capacity in participating countries [86].

#### Global Outbreak Alert and Response Network (GOARN) Training

Managed by WHO, GOARN is a global technical partnership that provides specialized training and rapid expert deployment during outbreaks [87]. Through extensive engagement and collaboration with more than 250 institutions, GOARN’s training initiatives, launched in 2005, provide specialized training in outbreak investigation, response, and coordination [88, 89]. GOARN has been critical in coordinating international responses to outbreaks like Ebola, H1N1 influenza, and COVID-19, providing a platform for rapid deployment and collaboration of trained experts worldwide [87, 90].

#### Global Laboratory Leadership Program (GLLP)

The GLLP developed through a collaboration between multiple global health organizations, plays a critical role in strengthening the leadership and management capabilities of laboratory professionals. Launched in 2019, GLLP focuses on building a workforce that is capable of effectively leading national and regional laboratories in outbreak detection and response efforts [91]. A foundational achievement of the GLLP is the development of the Laboratory Leadership Competency Framework to serve as a global standard for laboratory leadership development [92]. By fostering skills in laboratory systems management, biosecurity, and risk communication, the program has been implemented in at least 8 countries and ensures that laboratory leaders are well-prepared to meet the challenges of public health emergencies [92].

### Engage Communities

Community engagement can be defined as “a process of developing relationships that enable stakeholders to work together to address health-related issues and promote wellbeing to achieve positive health impact and outcomes” [93]. Successful community engagement requires the active participation of community members in program design, leadership, implementation, and monitoring and evaluation [93, 94]. A generic model and novel methodology for guiding earlier outbreak detection identified local recognition as the critical first step in disease detection [95]. Engaged communities play a pivotal role in the detection and management of epidemics.

#### Community Health Workers as a Cornerstone for Early Detection of Outbreaks

Most outbreaks often originate within communities and are frequently detected too late. Since communities are typically the first to experience the impact of epidemics, they play a crucial role in identifying early signs of an outbreak [96]. Community health workers (CHWs) are essential to early outbreaks detection. CHWs are often the first to observe unusual patterns of illness or symptoms that may signal the onset of an outbreak. Their close ties with community members enable them to detect and report early signs of diseases, and act as an essential early warning system for disease outbreaks [97].

CHWs, despite their underutilization, possess the potential to enhance primary care quality and accessibility. A review of thirty studies revealed twelve CHW’s functions—health coaching, social support, health assessment, case management, medication management, follow-up, administration, health education, and literacy support—as well as three key roles: clinical services, links to community resources, and health coaching and education [98].

The rapid spread of Ebola across geographic boundaries highlighted the urgent need for interventions capable of swiftly detecting, reporting, and treating cases at the community level. In response, trained CHWs played a crucial role in identifying cases, reporting alerts, and isolating individuals. In Sierra Leone, these initiatives significantly enhanced early case detection, although they also led to a number of false alerts [94, 99]. Despite this, the efforts of CHWs were instrumental in managing the outbreak and improving the overall response.

#### Community Based Surveillance

To improve the early detection of outbreaks and to sustain disease surveillance, early warning, alert, and response systems (EWARS) are recommended. Community-based surveillance (CBS) can be an important component of early warning systems. CBS has been defined as “the systematic detection and reporting of events of public health significance within a community by community members” [100]. The CBS workforce often include CHWs, healthcare workers and volunteers (teachers, faith leaders…) who are often frontline responders to detect and monitor health events in the community [101]. CBS implementation has benefited from advances in electronic tools for surveillance and digital health initiatives. One example of a promising digital health tool is the CommCare platform, specifically designed for large-scale implementation in LMICs; it enhances frontline healthcare delivery by streamlining data entry and enabling real-time monitoring [102, 103]. Several other innovative digital tools have been deployed to enhance the effectiveness of CHWs in managing infectious diseases outbreaks-related tasks and delivering essential health services. Notable among these are Living Goods’ Smart Health app, mHero, and Medic Mobile’s Community Health Toolkit [104–106]. CBS implementation experiences in Indonesia, Sierra Leone, and Uganda demonstrated that alerts generated by volunteers were highly accurate, matching community case definitions in 96 percent of cases in Sierra Leone, 90 percent in Indonesia, and 73 percent in Uganda. In Bangladesh, CBS, integrated with EWARS for five epidemic-prone diseases and community mortality, reported promising results for valid data and timely detection and response during a period of cholera and diphtheria outbreaks [107]. CBS is essential for early outbreak detection but faces challenges, including high false positives, inconsistent data quality, and limited diagnostic capabilities [108]. CBS also struggles with sustainability and scalability, as it often depends on external funding and volunteer efforts [45].

### Strengthen Governance

Public health governance is critical in ensuring nations are prepared to detect and respond to infectious disease threats effectively. WHO defines governance as ‘a wide range of steering and rulemaking related functions carried out by governments as they seek to achieve national health policy objectives’ [109]. When examining the various factors that enable early outbreak detection in low- and middle-income countries, governance emerges as a critical determinant [95]. Effective governance frameworks enable countries to establish robust systems for diagnostic and surveillance, stakeholder collaboration, and data sharing. WHO provides guidelines for developing national outbreak preparedness and response plans. These guidelines emphasize the importance of having clear leadership, defined roles and responsibilities, and effective coordination mechanisms at all levels of government [110]. Countries with well-established public health governance frameworks are better equipped to develop comprehensive outbreak preparedness and response plans. For example, during the 2014–2016 Ebola outbreak in West Africa, countries with stronger governance structures, such as Nigeria, were able to contain the virus more effectively. Nigeria’s success was attributed to its rapid activation of their emergency operations center, effective coordination among health authorities through their incidence management system, and strong political commitment to outbreak response [111]. This underscores the importance of governance in ensuring that outbreak response measures are implemented swiftly and effectively.

#### Stakeholder and International Collaboration

Stakeholder collaboration is another cornerstone of public health governance. Involving government agencies, non-governmental organizations, the private sector, and international partners is essential for mobilizing resources, sharing information, and coordinating responses. Since infectious diseases know no borders, international cooperation becomes crucial. Governance frameworks must facilitate data sharing, cross-border coordination, and access to financial and technical resources as mandated by the IHR [112, 113]. The One Health approach further underscores the need for robust governance. Establishing a governance framework is crucial for supporting One Health initiatives, such as the development of integrated surveillance systems capable of detecting potential outbreaks at the animal-human-environment interface [114, 115].

## Discussion

### Challenges and Opportunities in Implementing Timely Outbreak Detection Systems

#### Foundational Infrastructure for Diagnostics

Timely outbreak detection still hinges on the speed and reach of laboratory confirmation. Limited bench capacity, reagent shortages, and slow specimen referral create the first and often longest bottleneck in the 7-1-7 timeline [6, 116]. During West Africa’s 2014 Ebola outbreak, Guinea lacked local diagnostic capacity, and it took almost 3 months for health authorities and their international partners to confirm that the Ebola virus was the causative agent [6]. This delay allowed the virus to spread unchecked, far overshooting the 7-day detection goal. More broadly, a review of African cholera outbreaks reported to WHO (1996–2014) found a median delay of ∼27 days from a first case to outbreak detection, and a median of 7 days from detection to the start of investigation [117]. These figures underscore how late detection and slow investigation, often due to suboptimal real-time surveillance and laboratory networks, have hindered 7-1-7 benchmarks.

New DNO tools and regional genomic hubs now provide concrete opportunities to shorten this gap. Modelling studies in Kenya using DNO tools have shown that strategically placing district-level GeneXpert or PCR platforms can reduce sample transport times by as much as 40%, thereby significantly speeding up laboratory confirmation and outbreak detection [116]. Scaling these upgrades requires parallel investment in robust specimen-transport systems, quality-management schemes, and biosafety training—otherwise even the best equipment will sit idle [39–41]. Once laboratories can confirm signals quickly, the next hurdle is getting those signals into an integrated surveillance stream.

#### Data Integration: From Community Signals to National Dashboards

EBS and IBS often run on separate paper trails, delaying signal triage and blurring accountability [44, 48]. Countries such as Nigeria and Côte d’Ivoire have demonstrated that open-source platforms (e.g., SORMAS) can fuse EBS alerts, IBS line-lists, and laboratory results in near-real time, cutting duplicate data entry and reducing detection-to-notification intervals by one to two days [118, 119]. Interoperability standards, Open HIE architecture, and DHIS2 APIs—are now widely available and endorsed by WHO-AFRO [44]. However, experience shows that without multi-sectoral data-governance agreements (human, animal, and environmental health), platforms remain siloed and underused. Countries applying the WHO-AFRO IDSR strategy are increasingly aligning surveillance, laboratory, and digital systems under unified national health information exchange frameworks [45]. Multisectoral coordination mechanisms, such as emergency operations centers (EOCs), national public health institutes (NPHIs), and One Health platforms, play an essential role in harmonizing data flows between human, animal, and environmental health sectors.

Even with integrated data feeds, skilled people are required to interpret anomalies and initiate actions.

#### Human Capital: Workforce Numbers, Skills, and Retention

African public-health agencies face a triple workforce deficit: insufficient headcount, skills gaps, and high attrition [31, 120]. Health personnel shortages, often exacerbated by migration to higher income countries, are systemic problems [32, 120]. Inadequate education, poor working conditions, and a lack of continuous professional development led to skill gaps, low morale, and high turnover rates [32, 120]. These workforce issues directly affect the timeliness and effectiveness of outbreak detection. Graduates of programs such as FETP, OHW-NG, and GLLP are filling critical roles, yet overall epidemiologist density in sub-Saharan Africa remains only ∼3–5 per million population, roughly one-third of the WHO’s recommended benchmark of 10–15 field epidemiologists per million (Ref). Practical solutions include service-bonded training scholarships, tiered career ladders, and regionally pooled surge rosters [87, 89]. In Uganda, for example, a well-resourced emergency operations center and trained community surveillance officers have enabled timely outbreak response [121]. During a cholera outbreak at a border point in 2024, authorities detected, notified, and responded to the outbreak within the 7–1–7 timelines [121]. This rapid containment was explicitly attributed to the presence of a functional EOC and skilled frontline health workers [121]. Sustaining this skilled workforce and the systems they operate depends on governance, financing, and partnerships.

#### Governance, Financing, and Multi-Sector Coordination

Persistent governance challenges, including corruption, political instability, limited financial resources, continue to undercut detection performance [115, 117]. Access to funding is crucial for effective outbreak detection and response, as it enables governments to invest in essential diagnostic infrastructure, workforce training, and surveillance systems. By leveraging global initiatives like the World Bank’s Pandemic Fund, countries can secure the necessary resources to advance these capabilities, ensuring they have the tools and expertise needed to build resilient health systems and respond swiftly to emerging threats. The World Bank’s Pandemic Fund is designed to support countries in improving their health security by funding initiatives that enhance diagnostic networks, surveillance systems, and workforce development [122]. The United States (US) supported global health security initiatives were pivotal in bolstering infectious disease detection and surveillance capacity in low- and middle-income countries. Over the past two decades, the US emerged as the largest government donor in this field, providing financial aid and technical assistance that helped more than 30 countries strengthen their ability to prevent, detect, and respond to disease outbreaks [123]. Through major initiatives like the Global Health Security Agenda (GHSA) and targeted programs such as the President’s Emergency Plan for AIDS Relief (PEPFAR), U.S. investments enhanced laboratory diagnostics and disease surveillance systems in many countries [124, 125]. These efforts focused on building local expertise, optimizing diagnostic networks, and integrating surveillance data, which enabled faster outbreak detection and more effective epidemic responses–ultimately strengthening global health security [126].

Today, funding for many of these U.S.-funded programs has been scaled back or cut and the loss of U.S. support risks eroding progress. The abrupt halt of USAID assistance in late 2024 is projected to undermine outbreak response efforts in many African countries [126].

To safeguard and build on these gains, African governments and regional institutions now face urgent pressure to step up domestic investment in health security. Stronger local commitment is essential; only two African countries currently meet the Abuja target of allocating 15% of the national budget to healthcare–underscoring the need for others to boost funding in order to maintain resilient disease surveillance and response systems [127].

Public-private partnerships (PPPs), and sustained funding can significantly enhance outbreak detection capabilities. PPPs can provide additional resources, expertise, and technology transfer, facilitating the implementation of advanced surveillance tools and diagnostic networks. In partnership with Illumina and Oxford Nanopore, who provide essential in-kind contributions such as next-generation sequencing machines and training, the Africa Pathogen Genomics Initiative, spearheaded by the African Union Commission through Africa CDC, aims to enhance access to advanced genomic sequencing tools and expertise [128]. By pooling resources, expertise, and technology from both sectors, PPPs can facilitate the implementation of advanced surveillance tools and strengthen diagnostic networks. For instance, the Coalition for Epidemic Preparedness Innovations (CEPI) exemplifies how global collaboration can support African countries by providing financial and technical assistance for outbreak preparedness and response [129]. Finally, innovative technologies can amplify these investments, if implementation challenges are tackled head-on.

#### Technology Acceleration: AI, Genomics, and Wastewater Surveillance

AI-enhanced surveillance systems and genomic-surveillance networks promise earlier diseases detection, yet bias, data-sparsity, and compute costs limit routine use in many African countries [122, 123]. AI-enhanced surveillance offers promising advancements in early outbreak detection by enabling rapid analysis of large, heterogeneous datasets. However, implementing these tools is challenged by multiple systemic barriers. Limited digital infrastructure, insufficient funding, and a shortage of skilled personnel hinder the effective deployment and sustainability of such technologies in many African countries [130]. AI tools require robust data management systems, reliable internet connectivity, and continuous personnel training—all of which are frequently underdeveloped in low-resource settings [130].

In addition to these structural constraints, several technical and methodological challenges must also be addressed. One significant issue is data bias; AI and ML algorithms depend heavily on the quality and representativeness of input data [118]. Furthermore, computational demands for real-time data processing and model training can exceed the capacities of local systems, necessitating cloud-based solutions that may raise concerns around data security, sovereignty, and cost [118]. Similarly, WBE has the potential to generate the most reliable early-warning signals when laboratories have the molecular capacity, typically reverse-transcription quantitative PCR (RT-qPCR) to detect and quantify viral RNA in sewage with high analytical sensitivity [119, 131]. WBE requires high-throughput sample processing and the use of optimized protocols for pathogen concentration, recovery efficiency, and internal quality controls. To ensure result comparability across sites, standardized assays must be implemented, supported by trained laboratory personnel, robust quality management systems and biosafety [132]. Where these foundations are weak, as in many African countries, WBE implementation value as an early-warning tool is diminished.

Addressing these five interlocking themes in a coordinated manner is essential for African nations to consistently reach the 7-day detection milestone of the 7-1-7 framework. Integrated investment across laboratory capacity, data interoperability, workforce, governance, and targeted technology can collectively shrink Africa’s average detection delay—from weeks to days—and move the continent closer to universal compliance with the 7-1-7 targets.

### Recommendations

Achieving the first “7” in the 7-1-7 framework: detecting outbreaks within 7 days, requires coordinated and sustained investments across five critical pillars. First, countries must strengthen diagnostic networks to enable rapid, decentralized pathogen confirmation. Strategic placement of molecular platforms such as GeneXpert or RT-qPCR at the district level, coupled with improved specimen transport systems, can significantly reduce delays in confirmation and reporting. Second, integrating surveillance systems is essential. Data from community event-based surveillance must be rapidly linked with indicator-based systems through interoperable digital platforms that allow for seamless data sharing between community health workers, laboratories, and public health authorities. This integration ensures that early signals are validated and investigated without delay.

Third, a well-trained and retained workforce is critical for real-time detection and response. Investments should expand existing field-based training programs like FETP and GLLP, while creating incentives for retention and career progression within national health systems. Fourth, digital innovation—including AI and machine learning tools—should be leveraged to enhance the sensitivity and speed of outbreak detection. However, implementation must be context-specific and supported by adequate infrastructure and data governance frameworks. Lastly, effective governance and multisectoral coordination are foundational. National public health emergency operations centers should be empowered to coordinate across sectors using integrated data systems. Together, these actions reinforce early detection capabilities and support countries in consistently meeting the first critical milestone of the 7-1-7 framework.

## Conclusion

Timely outbreak detection remains a formidable but achievable goal for African countries. The 7-1-7 framework offers a powerful tool to monitor and improve detection timelines, but reaching its targets demands systemic improvements across diagnostics, surveillance, governance, and workforce development. While funding from global partners has enabled significant gains, sustaining momentum will require stronger domestic investment, multisectoral coordination, and iterative learning from past outbreaks. The integration of AI-enhanced surveillance, laboratory network optimization can support more resilient systems. Countries that align their national strategies with these innovations and build capacity across all levels, community to national, are better positioned to detect and contain outbreaks before they escalate. The path forward must be data-driven, inclusive, and country-owned. Achieving the first target: detecting outbreaks within 7 days, is not merely aspirational but essential for global health security.
